# AoChk1 Is Required for Sporulation, Trap Formation, and Metabolic Process in *Arthrobotrys oligospora*

**DOI:** 10.3390/jof11080602

**Published:** 2025-08-19

**Authors:** Huan Luo, Qianqian Liu, Si Chen, Xiaoli Li, Haitao Chen, Yuanyuan Xia, Jinkui Yang

**Affiliations:** State Key Laboratory for Conservation and Utilization of Bio-Resources, Key Laboratory for Microbial Resources of the Ministry of Education, School of Life Sciences, Yunnan University, Kunming 650091, China; luohuan@stu.ynu.edu.cn (H.L.); liuqianqian614@163.com (Q.L.); cs@mail.ynu.edu.cn (S.C.); lixiaoli@stu.ynu.edu.cn (X.L.); chenhaitao@stu.ynu.edu.cn (H.C.); xiayuanyuan@stu.ynu.edu.cn (Y.X.)

**Keywords:** serine/threonine protein kinase, asexual development, trap formation, secondary metabolism, pathogenicity

## Abstract

Chk1, a highly conserved serine/threonine protein kinase, functions as a critical regulator of fungal cell cycle progression, mitotic fidelity, and DNA damage response. In this study, we characterized an orthologous Chk1 (AoChk1) in a ubiquitous nematode-trapping fungus, *Arthrobotrys oligospora,* through targeted gene knockout coupled with integrated phenotypic, metabolomic, and transcriptomic analyses. This study aims to elucidate the function and potential regulatory networks of AoChk1 in *A. oligospora*. Deletion of *Aochk1* leads to significant reductions in nucleus number, hyphal cell length, conidial production, and trap formation, but an increase in the accumulation of lipid droplets and autophagy. In addition, transcriptomics data indicate that AoChk1 plays an important role in cell cycle and division, nuclear architecture and organelle dynamics, protein homeostasis maintenance, and membrane systems. In addition, the inactivation of the *Aochk1* exhibited remarkably reduced metabolite abundance relative to the WT strain. In conclusion, our results identify AoChk1 as an important regulator of asexual development, pathogenicity, and metabolic processes in *A. oligospora*.

## 1. Introduction

Cell cycle checkpoint kinase 1 (Chk1), an evolutionarily conserved serine/threonine protein kinase initially characterized in *Schizosaccharomyces pombe*, has been subsequently identified through comparative genomic analyses as an ubiquitously expressed regulatory protein across multiple eukaryotic taxa, including mammalia (murine and human models), diptera (*Drosophila melanogaster*), anura (*Xenopus laevis*), and other phylogenetically diverse organisms [1,2,3,4,5,6]. Biochemical and genetic studies have further established its critical role in maintaining genomic integrity through coordinated regulation of cell cycle progression and DNA damage response pathways [7,8]. Chk1 exhibits a canonical tripartite architecture comprising the following: (1) an *N*-terminal kinase domain demonstrating significant evolutionary conservation, featuring a catalytically competent active site with a conserved ATP-binding pocket and phosphotransferase activity essential for substrate phosphorylation-mediated signal transduction; (2) a central scaffold region; and (3) a C-terminal regulatory module containing conserved SQ/TQ cluster domains that serve as phosphorylation-dependent regulatory motifs [9,10,11]. Chk1 demonstrates evolutionarily conserved functionality across diverse eukaryotic species [12], and it functions as a DNA damage checkpoint kinase that senses genomic insults and replication stress, initiating cell cycle arrest at the G2/M transition to ensure proper DNA repair execution [13,14,15]. Chk1 regulates cell cycle progression by interacting with key molecules, including Cdc25 and Wee1, during DNA damage, modulating cyclin-dependent kinase activity to maintain cell cycle integrity [16,17,18]. Chk1 ensures mitotic fidelity to prevent chromosome missegregation and genomic instability [9,19,20,21,22].

The function of Chk1 orthologs has been characterized in several yeasts and filamentous fungi. In *Schizosaccharomyces pombe*, phosphorylation of the mediator protein Crb2 induces direct complex formation with Chk1, enabling its targeted recruitment to DNA double-strand break sites. This phosphorylation-dependent interaction constitutes an essential molecular checkpoint for DNA damage surveillance and repair fidelity [23]. Furthermore, emerging studies demonstrate that Chk1 orchestrates critical regulatory functions in fungal developmental processes, conidiation, stress adaptation, and virulence. In *Candida albicans*, Chk1 mediates osmotic stress adaptation by phosphorylating downstream response regulators to activate the Hog1 MAPK signaling pathway [24]. It transduces oxidative stress signals by phosphorylating Ypd1-Ssk1, thereby activating the cAMP-PKA pathway [25], and coordinates with its downstream response regulator Skn7 to regulate the expression of catalase and superoxide dismutase, thus mitigating ROS-mediated damage [26]. It is also essential for fungal cell wall integrity, regulating the expression of glucan synthase and mannosyltransferase through the downstream response regulator protein Ssk1 and the Hog1 MAPK signaling pathway [27]. In *S. pombe*, *wat1*Δ*chk1*Δ double mutants exhibit severe viability defects and elevated spontaneous recombination frequencies relative to single mutants. Cells lacking checkpoint kinase Chk1 display marked growth impairment, culminating in synthetic lethality [28]. In *Cochliobolus heterostrophus*, the causal agent of leaf blight in southern corn, *chk1* ablation induces aerial hyphae hypoplasia, autolysis, and conidiation deficiency [29]. In *Neurospora crassa*, coordinated epigenetic modifications of *chk1* and *chk2* maintain rhythmic *frq* transcription and safeguard circadian homeostasis under genotoxic stress [30]. In *Cryptococcus neoformans*, Chk1 regulates dNTP supply by activating ribonucleotide reductase (RNR), which is essential for DNA damage repair and DNA synthesis under replication stress [31]. This mechanism coordinates the DNA damage checkpoint to maintain genome stability and genetically interacts with RRM1 (the large subunit of RNR) to mediate stress adaptation responses [32].

Nematode-trapping fungi are microorganisms that utilize nematodes as nutrient sources through parasitic and trapping mechanisms under nutrient-limited conditions, serving as biological regulators of nematode population dynamics [33]. Nematode-trapping fungi exhibit significant adaptive plasticity, dynamically modulating their trophic strategies (saprotrophic to predatory transitions) in response to environmental resource availability [34]. Under nutrient-replete conditions, nematode-trapping fungi exist in a saprophytic phase, whereas under nutrient deprivation or in the presence of nematodes, their hyphae differentiate into specialized trapping apparatuses (traps)—including three-dimensional networks, adhesive branches, adhesive knobs, and constricting rings—to capture and digest nematodes [35,36,37]. Nematode-trapping fungi exhibit ubiquitous distribution, thus holding significant research relevance and biotechnological potential. *A. oligospora*, a representative species of nematode-trapping fungi, develops adhesive three-dimensional mycelial networks (traps) upon nematode or chemical induction to ensnare nematodes [38]. It is a commonly utilized model fungal organism employed to study nematode–fungal interactions [39]. In recent studies, several signaling pathways, including the cAMP-PKA signaling pathway [40,41], the MAPK signaling cascade [42,43,44], and G protein signaling [45], have been proven to modulate mycelial growth, trap formation, and pathogenicity. In this study, a homologous Chk1 (AoChk1) was retrieved from the fungus *A. oligospora*, and its functions were characterized through multi-phenotypic comparisons, metabolomic profiling, and transcriptomic assays. Our results demonstrate that AoChk1 plays pleiotropic roles in the conidiation, trap formation, and pathogenicity of *A. oligospora*.

## 2. Materials and Methods

### 2.1. Strains and Culture Conditions

The fungus *Arthrobotrys oligospora* (ATCC 24927) served as the wild type (WT), and the Δ*Aochk1* mutant strain was generated via homologous recombination [46]. *Escherichia coli* DH5α competent cells (Takara) were utilized as the host for plasmids pRS426 (cloning vector) and pCSN44 (harboring the hygromycin resistance gene *hph*) to maintain and propagate plasmid constructs. Recombinant vectors were selected using *Saccharomyces cerevisiae* FY834 on SC-Ura medium [47].

YPD (Yeast Extract Peptone Dextrose) medium was utilized for the cultivation of the yeast strain *S. cerevisiae* FY834 [47]. CMY (Corn dextrose with Yeast Extract) medium was used for spore production, and hyphal growth rates were assessed on PDA (Potato Dextrose Agar), TG (Tryptone–Glucose), and TYGA (Tryptone–Yeast Extract–Glucose Agar) media as previously described [48]. The nematode (*Caenorhabditis elegans*, N2 strain) was maintained on sterile oat medium (30 g/L oats, 60 mL ddH_2_O) at 26 °C.

### 2.2. Phylogenetic Tree Construction and Sequence Analysis

Phylogenetic analysis of AoChk1 (AOL_s00080g44) was performed by identifying its orthologs through BlastP searches against the *A. oligospora* genome using the Chk1 sequence (XKU26349.1) from the model fungus *S. cerevisiae*. The molecular weight (MW) and isoelectric point (pI) of AoChk1 were calculated via the ExPASy Compute pI/MW tool (http://web.expasy.org, accessed on 10 March 2025). Based on the amino acid sequences of Chk1 homologs in different fungi, the Neighbor-Joining (NJ) method was applied in MEGA 7.0 software to construct the phylogenetic trees [49,50]. At the same time, the Batch CD-Search tool was used on the NCBI website as well as TBtools software v2.148 to analyze the structural domains of the Chk1 proteins [51].

### 2.3. Deletion of Aochk1

The target gene *Aochk1* in *A. oligospora* was disrupted through a homologous recombination-mediated method [52,53]. Firstly, the genetic sequence of *Aochk1* was obtained from the NCBI database, and primers for the 5F and 3R ends of the target gene were designed using Primer 5 software. The target gene fragment was amplified via PCR using the designed primers (Table S1). The *hph* fragment was amplified using the pSCN44 plasmid as the template. The pRS426 vector, digested with *EcoRI* and *Xho*I, was transformed into the *S. cerevisiae* FY834 strain via PEG/CaCl_2_-mediated transformation. Recombinant strains were selected on SC-Ura medium, and the recombinant plasmid was harvested. Then, the replacement fragment was amplified using primers. Subsequently, the replacement fragment was transformed into the protoplasts of *A. oligospora*, and plated on PDAS medium supplemented with 200 μg/mL hygromycin B. Transformants were validated by PCR and real-time quantitative PCR (RT-qPCR) [14,54,55]. The primers used in this experiment are listed in Supplementary Table S1.

### 2.4. Assays of Mycelial Growth and Spore Yield

The WT strain and the mutant strains were inoculated onto PDA medium for 5 days of activation each. The uniformly sized clumps were obtained by punching holes from the outer rim of the mycelium using a sterile puncher. They were inoculated onto PDA, TG, and TYGA media at 28 °C in the dark, and the diameter of the colony was measured every 24 h until the 5th day [56].

Fungal mycelial blocks were inoculated onto CMY medium and incubated at 28 °C in the dark for 14 days. Mycelia were washed with 20 mL ddH_2_O, filtered, and resuspended to obtain a spore suspension. A 1 μL aliquot of the suspension was quantified under a microscope, with three biological replicates per group. Additionally, to compare spore germination rates, approximately 20,000 spores were spread onto WA plates and incubated at 28 °C in the dark. Spore germination rates were determined at 4, 8, and 12 h post-inoculation [57,58].

### 2.5. Comparison of Trap Formation and Pathogenicity

A spore suspension containing 20,000 spores of the WT and the mutant strain was spread evenly on WA medium and incubated at 28 °C for 3 days. Trap formation was induced by the addition of 200 nematodes, after which the number of traps and nematode lethality were observed and counted under a light microscope (Olympus, Tokyo, Japan) at 12, 24, 36, and 48 h [42,59,60].

### 2.6. Staining and Observation of Mycelial Structures

To observe the mycelial septa, the activated strains were incubated on PDA medium for 5 days, and the mycelia were stained using 20 μg/mL of calcium fluorescent white (CFW) (Sigma-Aldrich, St. Louis, MO, USA) and visualized under the DAPI (blue light) channel. The fungal mycelia were stained with 20 μg/mL of CFW and 20 μg/mL of 4′,6-diamidino-2-phenylindole (DAPI) (Sigma-Aldrich, D9542) to observe the nuclei as previously described [61]. Similarly, the lipid droplets (LDs) were visualized by staining with 10 μg/mL of boron dipyrrole methylene (BODIPY) (Thermo Fisher Scientific, Waltham, MA, USA). In addition, the autophagosomes were stained with 100 μg/mL of Monodansylcadaverine (MDC). The treated mycelia were observed using a fluorescence microscope. In addition, the LDs and autophagosomes were observed by transmission electron microscopy (TEM, Hitachi, Tokyo, Japan).

### 2.7. RT-qPCR Analysis

In order to detect the changes in the transcription levels of corresponding target genes, RT-qPCR was utilized. The activated mycelia were incubated on CMY medium lined with cellophane at 28 °C in the dark, and the mycelia were collected at 3, 5, and 7 days, respectively, and frozen with liquid nitrogen. The total RNA of the mycelia was extracted by using RNA Extraction Kit (Axygen Scientific, Union City, CA, USA); then, the RNA was reverse-transcribed into cDNA by using PrimeScriptTM RT reagent (Takara, Otsu, Shiga, Japan). The expression of genes related to sporulation was measured by RT-qPCR, with the β-tubulin gene (AOL_s00076g640) as an internal reference (Table S2) [14,54,62].

### 2.8. Analysis of Metabolomics

The activated mycelial block was inoculated into liquid PDB medium at 28 °C and 180 rpm for 7 days. The fermentation broth was collected, and 250 mL of ethyl acetate was added and mixed thoroughly. Then, the mixture was sonicated for 20 min, and this was repeated three times. The supernatant was extracted with a vacuum pump, and the extracts were solubilized with 1.5 mL methanol (chromatographic-grade ≥ 99%) and preserved in brown light-proof glass bottles. The sample was subjected to liquid chromatography–mass spectrometry (Thermo Fisher Scientific) and analyzed using Compound Discoverer 3.0 software [63,64,65,66,67].

### 2.9. Transcriptomics Analysis

To probe the regulatory influence of *Aochk1* on mycelial growth and trap formation in *A. oligospora*, comparative transcriptomic profiling was conducted between the WT and Δ*Aochk1* mutant strains. The fungal strains were inoculated onto a PDA plate at 28 °C for 5 days, and mycelia were harvested and induced with 800 nematodes for 0 and 24 h, respectively, in triplicate for each sample. All samples were frozen with liquid nitrogen and submitted for RNA sequencing by Genedenovo Biotechnology Co., Ltd. (Guangzhou, China). Bioinformatics analysis was performed using the OmicShare cloud platform (https://www.omicsmart.com/ accessed on 6 June 2025).

### 2.10. Data Analysis

Three biological replicates were performed for all data statistics, and all experimental data are expressed as the mean ± standard deviation (SD) of the three biological replicates. Data were analyzed using Prism 8.0 (Graph, San Diego, CA, USA) software using multiple *t*-tests, and statistically significant differences were indicated when *p* < 0.05.

## 3. Results

### 3.1. Bioinformatics Analysis and Knockout of the Aochk1 Gene

The *Aochk1* encodes a 595-amino acid polypeptide with a predicted MW of 66.45 kDa and a pI of 4.88. Phylogenetic analysis revealed that AoChk1 shares the highest sequence identity (90.91%) with its ortholog in nematode-trapping fungi *Arthrobotrys flagrans* within the same clade, high sequence similarities with other nematode-trapping fungi *Dactylellina haptotyla* (69.57%) and *Dactylellina cionopaga* (68.33%), and moderate similarities with other filamentous ascomycetes (e.g., *Aspergillus nidulans* 43.19%, *N. crassa* 40.32% and *S. cerevisiae* 31.73%). Structural domain annotation confirmed the presence of a PKC-like superfamily domain, demonstrating a high conservation across various fungi (Figure S1A).

Using the homologous recombination method, transformants were picked from the hygromycin resistance plate and verified using PCR and RT-qPCR. We finally obtained three mutants (Δ*Aochk1-3*, Δ*Aochk1-9*, and Δ*Aochk1-49*), which we used for subsequent experimental analysis (Figure S1B,C).

### 3.2. Aochk1 Gene Affects the Length of Mycelial Cells and the Number of Nuclei

**Figure 1:**
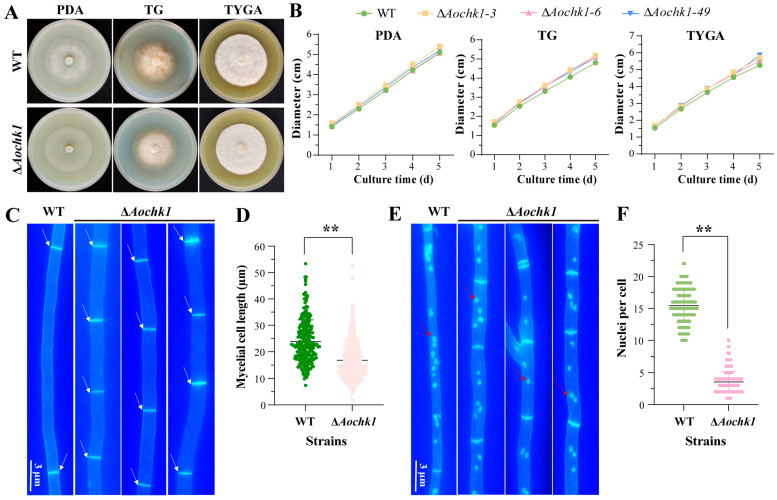
Comparison of hyphal growth rate and nuclear number between WT and Δ*Aochk1* mutant strains. (**A**) Colony morphology of WT and Δ*Aochk1* strains on different media. (**B**) Growth rates of WT and Δ*Aochk1* strains on PDA, TG, and TYGA media. (**C**) Hyphal septa in WT and Δ*Aochk1* strains, Bar = 3 μm, (white arrows indicate septa). (**D**) Hyphal compartment length of WT and Δ*Aochk1* strains. (**E**) Nuclear staining in hyphae of WT and Δ*Aochk1* strains, Bar = 3 μm, (red arrows indicate nuclei). (**F**) Quantitative comparison of nuclear number per hyphal compartment between strains. (Tukey’s HSD test: ** *p* < 0.01).

The activated WT and Δ*Aochk1* strains were cultured on PDA, TG, and TYGA media at 28 °C for 5 days. Colony diameter was measured daily to assess growth rate. The result revealed that *Aochk1* deletion did not alter mycelial growth rate (Figure 1A,B). Concurrently, CFW staining revealed a significant reduction in mean hyphal compartment length concomitant with increased septation frequency in the mutant strain relative to WT (Figure 1C,D). Furthermore, DAPI staining revealed a statistically significant reduction in mean nuclear count per mycelial cell in the mutant strain relative to WT, with WT exhibiting an approximately six-fold higher nuclear density (Figure 1E,F).

### 3.3. Deletion of Aochk1 Impairs Sporulation and Germination Rate

**Figure 2:**
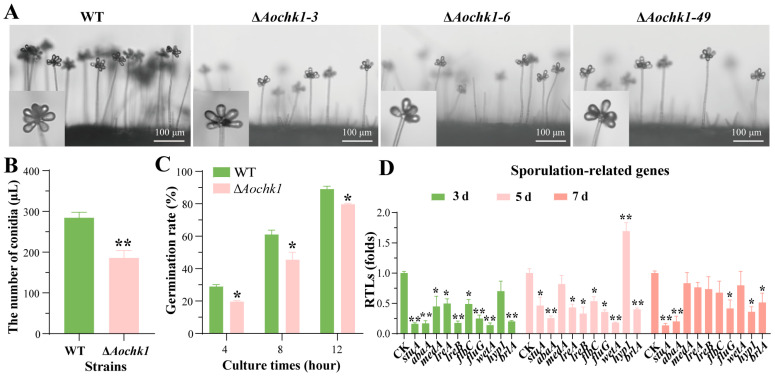
Comparative analysis of spore-related phenotypes between WT and Δ*Aochk1* mutant strains. (**A**) Representative images of spores produced by WT and Δ*Aochk1* strains after 3-day incubation on PDA medium, Bar = 100 μm. (**B**) Quantitative comparison of spore production. (**C**) Comparison of spore germination rates. (**D**) Relative expression levels of sporulation-related genes in WT and Δ*Aochk1* strains. (Tukey’s HSD test: * *p* < 0.05, ** *p* < 0.01).

To assess the role of *Aochk1* in conidiation, activated WT and Δ*Aochk1* strains were inoculated onto CMY medium at 28 °C for 14 days. The inactivation of *Aochk1* resulted in a remarkable reduction in the number of conidiophores (Figure 2A). Microscopic quantification (1 μL aliquot) revealed that the Δ*Aochk1* mutant exhibited reduced conidial yield, with nearly 100 fewer spores per microliter than the WT strain (Figure 2B). In addition, the spore germination rate of the Δ*Aochk1* mutant was decreased relative to the WT strain (Figure 2C). Meanwhile, the transcription levels of most spore-producing-related genes, including the central regulatory genes *abaA*, *wetA*, and *brlA*, were remarkably downregulated in Δ*Aochk1* mutant on the third and fifth days (Figure 2D). Therefore, *Aochk1* ablation significantly impairs sporulation capacity and compromises spore viability.

### 3.4. Aochk1 Is Involved in Regulating Trap Formation and Pathogenicity

**Figure 3:**
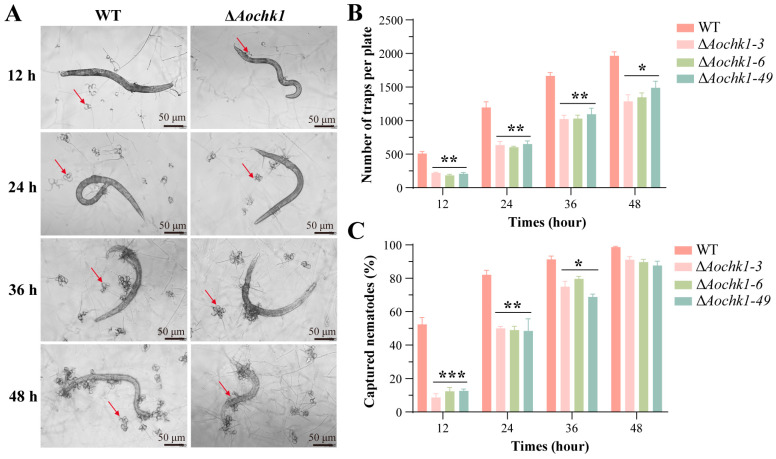
Comparative analysis of trap formation and predation ability. (**A**) Representative images of nematode predation by traps following nematode induction, Bar = 50 μm, (arrows indicate traps). (**B**) Comparison of trap formation in WT and Δ*Aochk1* strains. (**C**) Pathogenicity assay of WT and Δ*Aochk1* strains against nematodes. (Tukey’s HSD test: * *p* < 0.05, ** *p* < 0.01, *** *p* < 0.001).

Trap morphogenesis is a critical step for the nematode predation and lifestyle transition of *A. oligospora*. To further investigate the role of *Aochk1* in trap formation and pathogenicity, the trap formation was observed and quantified after the WT and Δ*Aochk1* mutant strains were induced in *C. elegans*. The results showed that the mutant strain exhibited a significant reduction in trap production and nematode predation efficiency compared to the WT (Figure 3A). Traps were formed in both WT and mutant strains at 12 h after induction, but the number of traps in the mutant strain was 50% lower than in the WT strain. This corresponded with markedly divergent nematocidal capacity; WT achieved 50% mortality versus 10% for the mutant. Although trap morphology showed no significant differences thereafter, the mutant consistently demonstrated fewer traps and substantially attenuated virulence at 24, 36, and 48 h post-induction (Figure 3B,C).

### 3.5. Transcriptomic Analysis of the Aochk1 Gene

**Figure 4:**
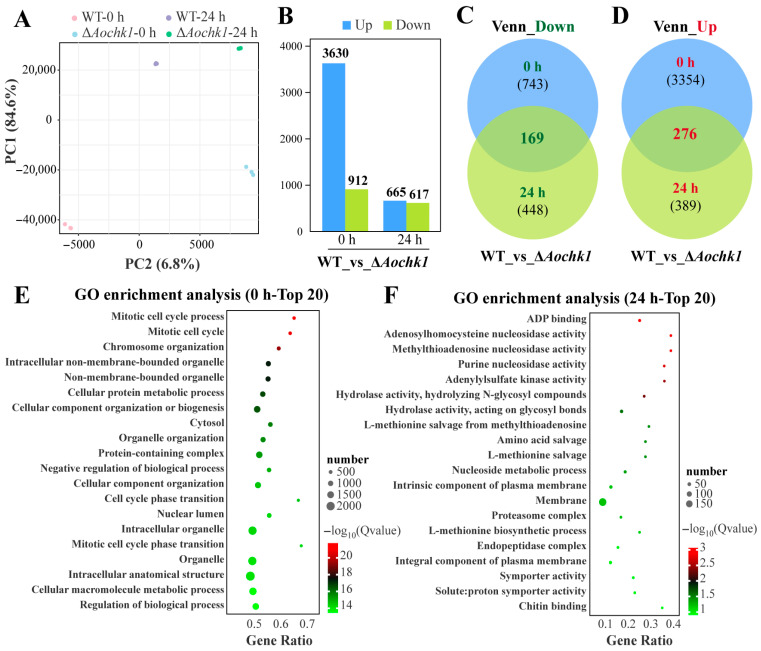
Transcriptomic profiling of the Δ*Aochk1* mutant strain. (**A**) Principal component analysis of transcriptomes from WT and Δ*Aochk1* mutant strains. (**B**) Bar plot showing the number of differentially expressed genes (DEGs). (**C**) Venn diagram of consistently downregulated DEGs shared at both 0 h and 24 h post-induction. (**D**) Venn diagram of consistently upregulated DEGs shared at both 0 h and 24 h post-induction. (**E**,**F**) Top 20 significantly enriched GO terms for DEGs identified at 0 h (**E**) and 24 h (**F**) (Δ*Aochk1* vs. WT).

To elucidate the potential mechanisms of *Aochk1*, genome-wide transcriptome profiling was conducted on Δ*Aochk1* mutants. Biological triplicates per sample yielded 37.27–44.72 million high-quality reads per library. Quality metrics demonstrated Q20 > 99.08%, Q30 > 96.89%, and GC content > 47.88% (Table S3). We further validated the transcriptome data through RT-qPCR analysis. First, we conducted cluster analysis on 21 related genes in the cell cycle, glycolysis/gluconeogenesis, and MAPK signaling pathways. The results showed that six related genes in the cell cycle pathway had high expression levels in Δ*Aochk1* mutants, while these genes showed low expression levels in the other two pathways (Figure S2). Additionally, principal component analysis revealed clear segregation of WT and mutant clusters across temporal phases, with high intra-group correlation confirming robust biological reproducibility for downstream analyses (Figure 4A). Transcriptomic profiling revealed substantial differential gene expression in Δ*Aochk1* versus WT. Under the vegetative growth stage (0 h), 3630 genes were significantly upregulated and 912 downregulated. Following a 24 h nematode challenge, these shifts attenuated to 665 upregulated and 617 downregulated differentially expressed genes (DEGs) (Figure 4B). Venn analysis robustly identified 169 persistently downregulated and 276 consistently upregulated DEGs common to both 0 h and 24 h timepoints (Figure 4C,D). To delineate *Aochk1*-mediated regulatory networks in *A. oligospora*, GO enrichment analysis of DEGs at 0 h and 24 h was performed. At the 0 h timepoint, significantly enriched terms were categorized into three functional modules: (1) cell cycle and division, involving mitotic cell cycle process, cell cycle phase transition, and mitotic cell cycle phase transition; (2) nuclear architecture and organelle dynamics, involving chromosome organization, nuclear lumen, intracellular non-membrane-bounded organelle, and organelle organization; and (3) protein homeostasis and regulatory systems, involving cellular protein metabolic process, protein-containing complex assembly, negative regulation of biological process, and regulation of biological process (Figure 4E). The experimental data shows that, at the 24 h timepoint, the main enriched GO terms can be categorized into the following groups according to their functional description: (1) hub for purine/thionucleoside metabolism, involving ADP binding, adenylylsulfate kinase activity, methylthioadenosine nucleosidase activity, and nucleoside metabolic process; (2) protein turnover system, involving hydrolase activity, endopeptidase complex, and amino acid salvage (Figure 4F).

We further analyzed KEGG enrichment of DEGs that were upregulated at 0 h. The enriched pathways were mainly classified by function as follows: (1) protein homeostasis maintenance, involving ribosome, proteasome, aminoacyl-tRNA biosynthesis, and RNA degradation; (2) DNA integrity assurance system, involving mismatch repair, DNA replication, homologous recombination, and base excision repair; (3) cell cycle and genetic information transfer, involving cell cycle, meiosis, and nucleocytoplasmic transport; and (4) metabolic reprogramming, involving pyrimidine metabolism and glycosphingolipid biosynthesis (Figure S3A). In the downregulated pathway at 0 h, we found an enrichment of a variety of amino acid metabolic pathways, such as purine metabolism, carbon metabolism, aspartate and glutamate metabolism, phenylalanine metabolism, aspartate and glutamate metabolism, arginine and proline metabolism. At the same time, the MAPK signaling pathway was also downregulated (Figure S3B). Further KEGG enrichment analysis of upregulated DEGs at 24 h demonstrated enrichment of the nitrogen metabolism, proteasome, fatty acid degradation, amino metabolism, ubiquitin-mediated proteolysis, glycerolipid metabolism, DNA replication, protein processing in endoplasmic reticulum, glycolysis/gluconeogenesis, and other upregulated pathways (Figure S3C). In the 24 h downregulated pathway, biosynthesis of secondary metabolites, nitrogen metabolism, fatty acid elongation, glycerophospholipid metabolism, sphingolipid metabolism, and various types of *N*-glycan biosynthesis were mainly enriched (Figure S3D).

Transcriptomic profiling of *A. oligospora* during the initial stages of nematode interaction identified 525 genes constitutively enriched at both 0 h and 24 h post-induction. Furthermore, comparative analysis revealed 757 DEGs were specifically enriched following nematode exposure, implicating their potential involvement in trap morphogenesis (Figure S4A). Gene Ontology (GO) enrichment analysis of these DEGs highlighted significant overrepresentation of terms associated with membrane biology. The GO terms that were enriched can mainly be categorized as follows: (1) Terminology for membrane systems includes intrinsic component of plasma membrane, intrinsic component of external side of plasma membrane, integral component of membrane, rough endoplasmic reticulum membrane, and side of membrane. These genes focus on transmembrane proteins and may be involved in related pathways such as cell membrane signaling or protein secretion. (2) Macromolecular complex terminology includes viral capsid, proteasome core complex, beta-subunit complex, DASH complex, outer kinetochore, mannan polymerase complex, and magnesium chelatase complex. (3) Terms related to enzyme activity include alpha-1,2-galactosyltransferase activity, aspartic-type, endopeptidase/peptidase activity, serine-type endopeptidase inhibitor activity, and glutamate dehydrogenase activity. These genes may be involved in glycosylation modifications and regulation of protein hydrolysis and key enzymes of amino acid metabolism (Figure S4B). Additionally, KEGG pathway analysis further demonstrated significant enrichment of DEGs in diverse metabolic pathways; these included amino acid metabolism (tyrosine metabolism, tryptophan metabolism, arginine biosynthesis, alanine, aspartate and glutamate metabolism), glycerolipid metabolism, nitrogen metabolism, various types of *N*-glycan biosynthesis, and biosynthesis of secondary metabolites (Figure S4C).

### 3.6. Aochk1 Is Involved in Regulating Lipid Metabolism and Autophagy Levels

**Figure 5:**
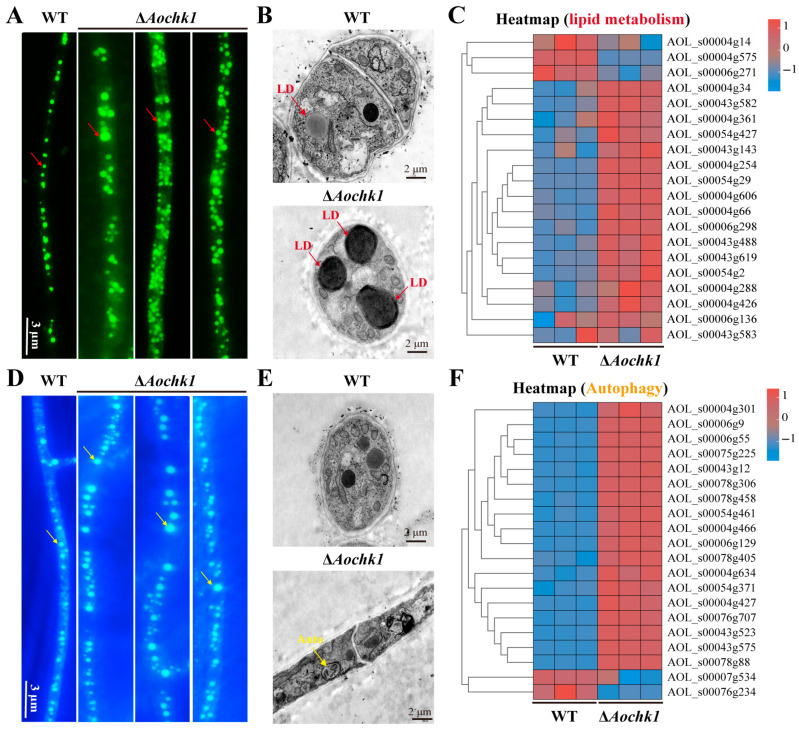
Comparison of lipid droplets (LDs) and autophagy between WT and Δ*Aochk1* mutant strains. (**A**) Staining of LDs in hyphae of WT and Δ*Aochk1* strains. Red arrows: lipid droplets. Bar = 3 µm. (**B**) Transmission electron microscopy (TEM) of intracellular LDs in WT and Δ*Aochk1* strains, Bar = 2 μm. (**C**) Cluster analysis of the expression levels of lipid metabolism-related genes in WT and Δ*Aochk1* mutant strains. (**D**) Autophagosomes in hyphae of WT and Δ*Aochk1* strains. Yellow arrows: autophagosomes. Bar = 3 µm. (**E**) Observation of autophagosomes by TEM, Bar = 2 μm. (**F**) Cluster analysis of the expression levels of autophagy-related genes in WT and Δ*Aochk1* mutant strains.

Intracellular LD distribution and morphology were assessed using BODIPY fluorometric staining. The WT hyphae exhibited uniformly dispersed LDs, whereas Δ*Aochk1* mutants displayed pronounced LD accumulation, as well as an increased number of LDs, and partial LDs had gathered together (Figure 5A). Ultrastructural observation via TEM also found LD accumulation, revealing a significant increase in both LD size and density within mutant hyphae relative to WT (Figure 5B). Clustering analysis of 20 lipid metabolism-associated genes showed that the transcripts of 17 genes were upregulated in the Δ*Aochk1* mutant, including the following: AOL_s00054g761 (serine palmitoyltransferase), AOL_s00004g426 (dihydroxyacetone kinase), AOL_s00006g298 (acyl-CoA-dependent ceramide synthase), AOL_s00043g488 (O-acyltransferase), AOL_s00043g619 (lysophospholipid acyltransferase, LPLAT), and AOL_s00004g254 (aldehyde dehydrogenase) (Figure 5C). Similarly, MDC staining revealed that increased autophagic vesicles were observed in Δ*Aochk1* hyphae compared to WT (Figure 5D). TEM observation also found autophagosomes in mutant cells (Figure 5E). Clustering analysis of 20 autophagy-associated genes showed that the transcript of 18 genes was upregulated, including the following: AOL_s00004g301 (autophagy-related protein 11), AOL_s00078g306 (serine/threonine-protein kinase), AOL_s00004g466 (vacuolar protein sorting Vps16), and AOL_s00078g405 (cAMP-dependent protein kinase A, Pka) (Figure 5F). Collectively, these findings demonstrate a central regulatory role for *Aochk1* in modulating lipid homeostasis and autophagic flux in *A. oligospora*.

### 3.7. Aochk1 Is Involved in the Regulation of Secondary Metabolism

**Figure 6:**
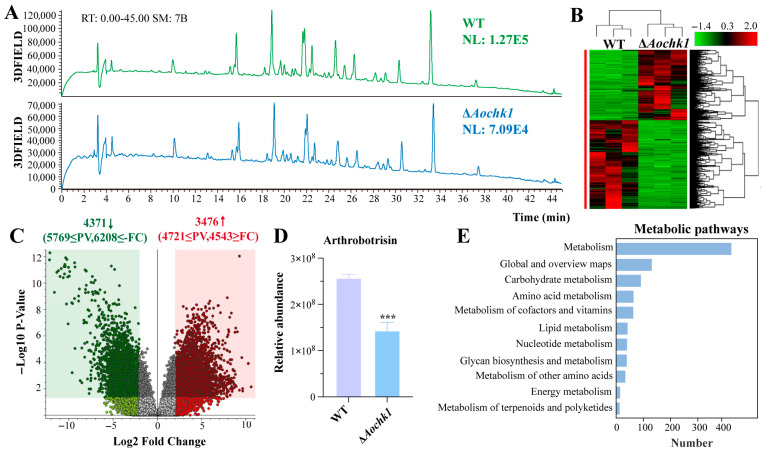
Comparative secondary metabolomic profiling of WT and Δ*Aochk1* mutant strains. (**A**) HPLC chromatograms of secondary metabolites extracted from WT and Δ*Aochk1* strains. (**B**) Clustering analysis of differential metabolites between strains. (**C**) Volcano plot of upregulated and downregulated metabolites. (**D**) Comparison of arthrobotrisin relative abundance between WT and mutant strain. (**E**) KEGG enrichment analysis of differential metabolites. (Tukey’s HSD test: *** *p* < 0.001).

Metabolome profiling reveals *Aochk1*-dependent regulation in *A. oligospora.* LC-MS analysis of extracts of culture filtrates revealed distinct secondary metabolite profiles between the WT and Δ*Aochk1* mutant strains. Although overall chromatographic peaks remained consistent, the Δ*Aochk1* mutant exhibited remarkably reduced metabolite abundance relative to the WT strain (Figure 6A). Differential features were predominantly localized to the 14–34 min retention time. Clustering of metabolite intensities confirmed widespread downregulation in the mutant strain (Figure 6B). Volcano plot analysis identified 4371 significantly downregulated and 3476 upregulated compounds in Δ*Aochk1* versus WT (Figure 6C). Notably, arthrobotrisins-specific metabolites were detected in both WT and mutant strains, with significantly reduced accumulation observed in the mutant compared to WT (Figure 6D). The differential metabolites were enriched in several KEGG pathways, such as carbohydrate metabolism, amino acid metabolism, metabolism of cofactors and vitamins, lipid metabolism, nucleotide metabolism, and glycan biosynthesis and metabolism (Figure 6E).

## 4. Discussion

Chk1 is an evolutionarily conserved serine/threonine protein kinase that plays a pleiotropic role in the growth and development of fungal cells by regulating the cell cycle, responding to DNA damage and replication stresses, and ensuring mitotic fidelity, and this function remains highly consistent from yeast to mammals [1,9,68,69,70]. In this study, we characterized the role of AoChk1 in the sporulation, trap formation, pathogenicity, LD accumulation, autophagy and secondary metabolism of *A. oligospora*. Our results suggested *Aochk1* is involved in various cellular processes and plays a crucial role in the proper development of nuclei, sporulation, lipid metabolism, and the nematode predation process.

It was shown that Chk1 orthologs are involved in mycelial growth and development and spore formation. In the corn leaf pathogen *C. heterostrophus*, the causal agent of southern corn leaf blight, *chk1* ablation induces aerial hyphae hypoplasia, autolysis, and conidiation deficiency, while the variety of phenotypic changes suggests that Chk1 is involved in multiple developmental pathways, each of which responds to different signals. When involved in multiple pathways, different components or even pathways may be required to assist in specific responses to specific signals [29]. In the ink cap mushroom *Coprinopsis cinerea*, deletion of *chk1* impairs fruiting body development; furthermore, they speculated that developmental defects were the result of cell cycle damage [71]. In the pathogenic yeast *Candida albicans*, Chk1 governs the transition from yeast to hyphal growth, a process critical for host invasion [72]. In our study, although deletion of *Aochk1* did not influence the mycelial growth, the ∆*Aochk1* mutant strains exhibited significant reductions in spore production, spore germination rate, and mycelial length compared to WT. Thus, we speculate that Chk1 participates in multiple developmental pathways, and the developmental defects of spores and changes in mycelial length are also the result of Chk1 causing cell cycle damage. Chk1 orthologs share multiple roles in vegetative growth and spore production in *A. oligospora* and other fungi.

In addition, Chk1 orthologs are involved in the pathogenic process of various fungi. In *Candida albicans*, *chk1* modulates virulence and cell wall biosynthesis, with its deletion compromising virulence factor expression and restricting hyphal development to a non-pathogenic form [73,74]. During the virulence program induction process of the plant pathogenic fungus *Ustilago maydis*, cell cycle arrest at the G2 phase is essential for the implementation of the virulence infection process, which promotes the development of infective hyphae. The activation of Chk1 is also triggered during this process, leading researchers to conclude that the Chk1 gene is crucial for pathogenicity and plays a key regulatory role in host colonization and disease development [75]. In *C. neoformans*, knockout of Rad53 and Chk1 promotes macrophage phagosome maturation, reduced melanin production, and increased sensitivity to oxidative stress, thereby reducing their pathogenicity [13]. In *C. heterostrophus*, *chk1* deletion results in severely reduced pigmentation, virulence, and conidiation [29]. In this study, deletion of *Aochk1* resulted in decreased trap production and depressed nematode predation ability. Additionally, based on transcriptomic data, the GO term obtained from the GO enrichment analysis of 757 differentially expressed genes can be categorized by function into the following three sections: membrane system-related terminology, macromolecular complex terminology, and enzyme activity-associated terms. This transcriptomic signature aligns with the established ultrastructural and biochemical events critical for trap functionality. Upon nematode contact, hyphal differentiation is initiated, culminating in the curling formation of specialized traps. This process necessitates extensive membrane remodeling, characterized by the localized secretion of adhesive macromolecules (e.g., polysaccharides, glycoproteins) to ensnare nematodes [76], followed by the targeted release of extracellular hydrolytic enzymes (e.g., proteases, chitinases) to penetrate the nematode cuticle and facilitate nutrient acquisition [77]. Consequently, membrane dynamics and protease activity constitute indispensable components of efficient trap formation and predation nematodes. Integrated analysis of the observed phenotypic alterations and the molecular profiling data strongly supports the conclusion that AoChk1 exerts a pivotal regulatory function within the signaling pathway governing nematode trap formation in *A. oligospora*. Therefore, Chk1 orthologs are conserved in the regulation of pathogenicity in *A. oligospora* along with other fungi.

It has been shown that decreased phospholipid availability is consistent with decreased Chk1 signaling and vice versa, and that an increase in phospholipid pools contributes to the protection of DNA integrity during genotoxic attacks [78]. In *A. oligospora*, compared with WT, LD accumulation was increased in the Δ*Aochk1* mutant strain, and by clustering analysis of DEGs, we found that the expression of many genes related to lipid metabolism underwent a significant upregulation. Meanwhile, we found a significant enrichment at 24 h in the glycerolipid metabolism in the upregulation pathway. The above results suggest that AoChk1 plays an important regulatory role in the lipid metabolism of *A. oligospora.*

Autophagy primarily functions as a cytoprotective mechanism. DNA damage induces autophagic activation, which plays a pivotal role in maintaining genomic stability [79,80,81]. In addition, autophagy is critically required for DNA repair by homologous recombination [81,82,83,84]. DNA damage triggers a series of signaling cascades that promote cell survival, including DNA repair, cell cycle arrest, and autophagy [85,86,87]. In our study, clustering analyses of the DEGs showed that the expression of 18 autophagy-related genes was significantly elevated. In addition, KEGG enrichment analysis of DEGs by transcriptomic data revealed that the homologous recombination, base excision repair, ribosome, and nucleocytoplasmic transport pathways underwent significant upregulation. Homologous recombination is a vital process for repairing DNA double-strand breaks [88], suggesting that deletion of *Aochk1* leads to DNA damage, which, in turn, triggers increased autophagy.

Chk1 is involved in the regulation of multiple metabolic pathways and the production of secondary metabolites in *A. oligospora*. In this study, through our combined transcriptomic and metabolomic analysis, we found that deletion of *Aochk1* led to changes in multiple metabolic pathways, such as carbohydrate metabolism, amino acid metabolism, metabolism of cofactors and vitamins, lipid metabolism, nucleotide metabolism, and glycan biosynthesis and metabolism. In addition, the content of arthrobotrisins was decreased in the Δ*Aochk1* mutant. Previous studies on Chk1 homologs in other fungi have primarily focused on mechanisms such as DNA damage response and cell cycle regulation, whereas their role in metabolic regulation remains understudied. Consequently, elucidating the metabolic regulatory function of Chk1 in *A. oligospora* provides novel insights for analogous research in other fungal systems.

Studies have shown that Chk1 responds to replication stress by regulating cell cycle checkpoints and homologous recombination repair [89]. In *N. crassa*, Chk1/2 is involved in the regulation of metabolism during DNA damage, modulation of chromatin structure, and robust, regular transcription of DNA repair genes [30]. In *S. pombe*, Crb2 recruits Chk1 to double-strand breaks for DNA damage repair by phosphorylation, thus maintaining DNA integrity in the genome [23], and Wat1 deletion induces chromosome breaks that activate the DNA damage checkpoint, and in *chk1*-null cells, this results in severe proliferation defects and synthetic lethality [28]. In *S. pombe*, Chk1 is important for maintaining the DNA replication checkpoint in heat-sensitive mutants in the S phase [90]. Synergistic or divergent control of RNR1 and RNR21 expression by Rad53 and Chk1 kinases was observed in response to DNA damage and DNA replication stress in *C. neoformans* [32]. In *C. cinerea*, the absence of Chk1 impairs the development of fruiting bodies, and downregulation of *chk1* and *Atr1* levels leads to mitotic abnormalities and a significant increase in cell number [71]. In this study, deletion of *Aochk1* significantly reduced the nuclear number. Transcriptome data shows that deletion of *Aochk1* results in alterations of cell cycle, mismatch repair, DNA replication, homologous recombination, meiosis, and other related pathways that are altered as a result of cell cycle dysregulation. The above results indicate that AoChk1 is a genome stability guardian, cell cycle engine regulator, and protein quality control system, and it has been shown that Chk1 is a key factor in the error-free DNA repair process of homologous recombination [87]. These findings suggest that the function of Chk1 in maintaining genomic integrity is relatively conserved in *A. oligospora* and other fungi.

## 5. Conclusions

This study comprehensively characterizes the functions of AoChk1 in *A. oligospora*, revealing its pivotal role in orchestrating diverse biological processes, including sporulation, trap formation, lipid metabolism, autophagic flux, and metabolic processes. Additionally, we identified previously the undescribed regulatory functions of Chk1 in other fungi, related to the number of nuclei, the formation of traps, and secondary metabolites (such as arthrobotrisins). Our findings provide novel insights into Chk1-mediated mechanisms in nematode-trapping fungi, facilitating the development of enhanced nematocidal agents and biocontrol strategies.

## Data Availability

The RNA-seq datasets produced in this study are archived in the NCBI Gene Expression Omnibus repository under accession GSE301452.

## Supplementary Materials

The following supporting information can be downloaded at: https://www.mdpi.com/article/10.3390/jof11080602/s1, Figure S1: Phylogenetic analysis of Chk1 orthologs and knockout validation of the *Aochk1* gene in *A. oligospora*; Figure S2: RT-qPCR analysis validated the transcriptome data of the wild-type and Δ*Aochk1* mutants. Figure S3: KEGG pathway enrichment analysis of differentially expressed genes (DEGs); Figure S4: Analysis of trap-associated transcriptomic data; Table S1: Primers used for genetic manipulation in this study; Table S2: List of primers used for RT-qPCR analysis in this study; Table S3: Statistical analysis of quality control data of 12 sequencing samples.
