# Supplementary material for: AoChk1 Is Required for Sporulation, Trap Formation, and Metabolic Process in Arthrobotrys oligospora
Source: J Fungi (Basel). 2025 Aug 19;11(8):602. doi: 10.3390/jof11080602 (PMC12387206; doi:10.3390/jof11080602)
Supplement: Supplementary file 1 [file jof-11-00602-s001.zip › jof-3780003-supplementary.pdf]

## Supporting Information

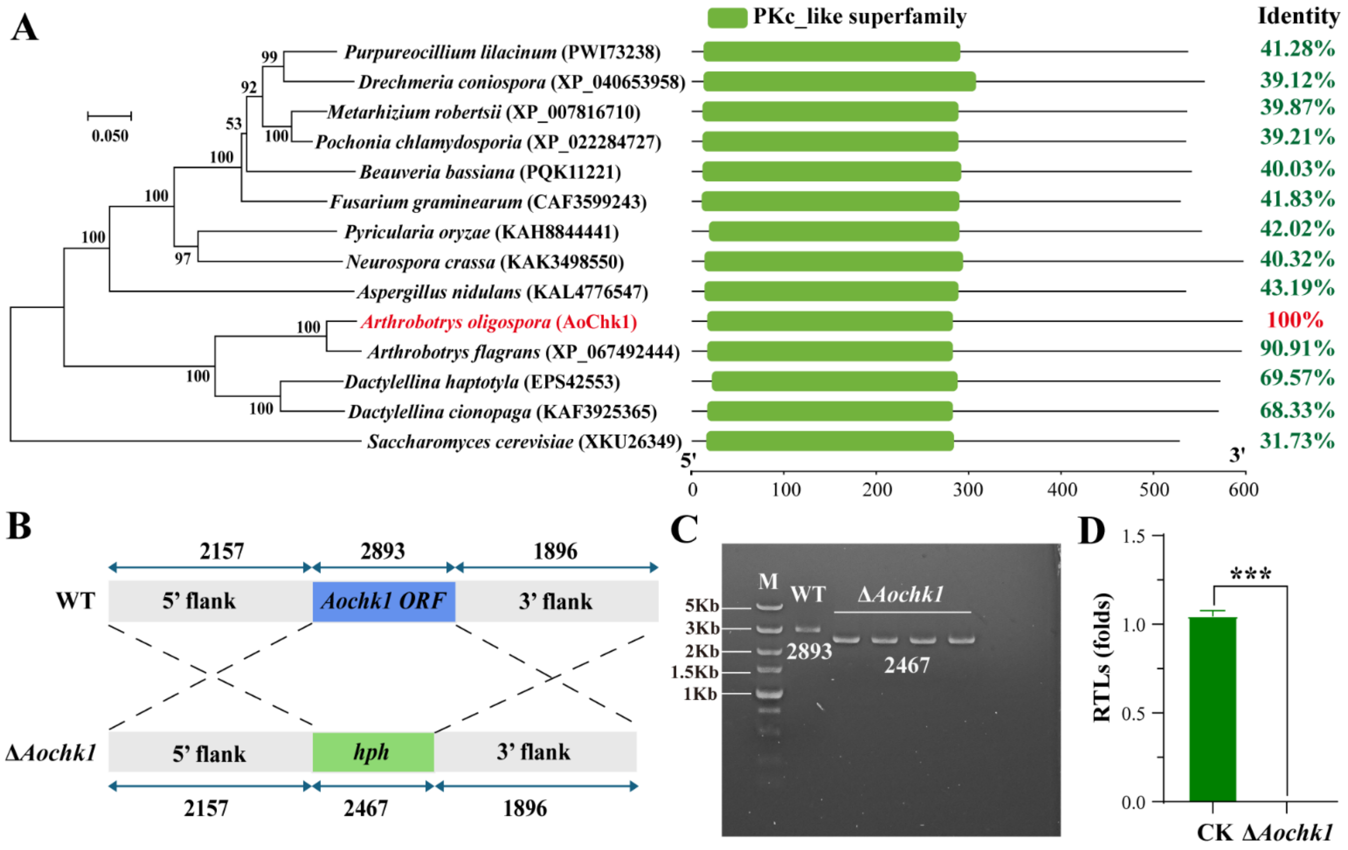


**Figure S1.** Phylogenetic analysis of Chk1 orthologs and knockout validation of the *Aochk1* gene in *A. oligospora*. (**A**) Phylogenetic tree of Chk1 homologs from diverse fungi (left panel), conserved functional domains of *Aochk1* orthologs (middle panel), and the sequence similarities of AoChk1 with orthologs (right panel). (**B**) Schematic diagram of the *Aochk1* gene knockout strategy via homologous recombination; (**C**) PCR verification of positive transformants for the *Aochk1* knockout mutants; (**D**) RT-qPCR validation of *Aochk1* gene deletion in positive transformants. (Tukey's HSD test: ***$p < 0.001$).

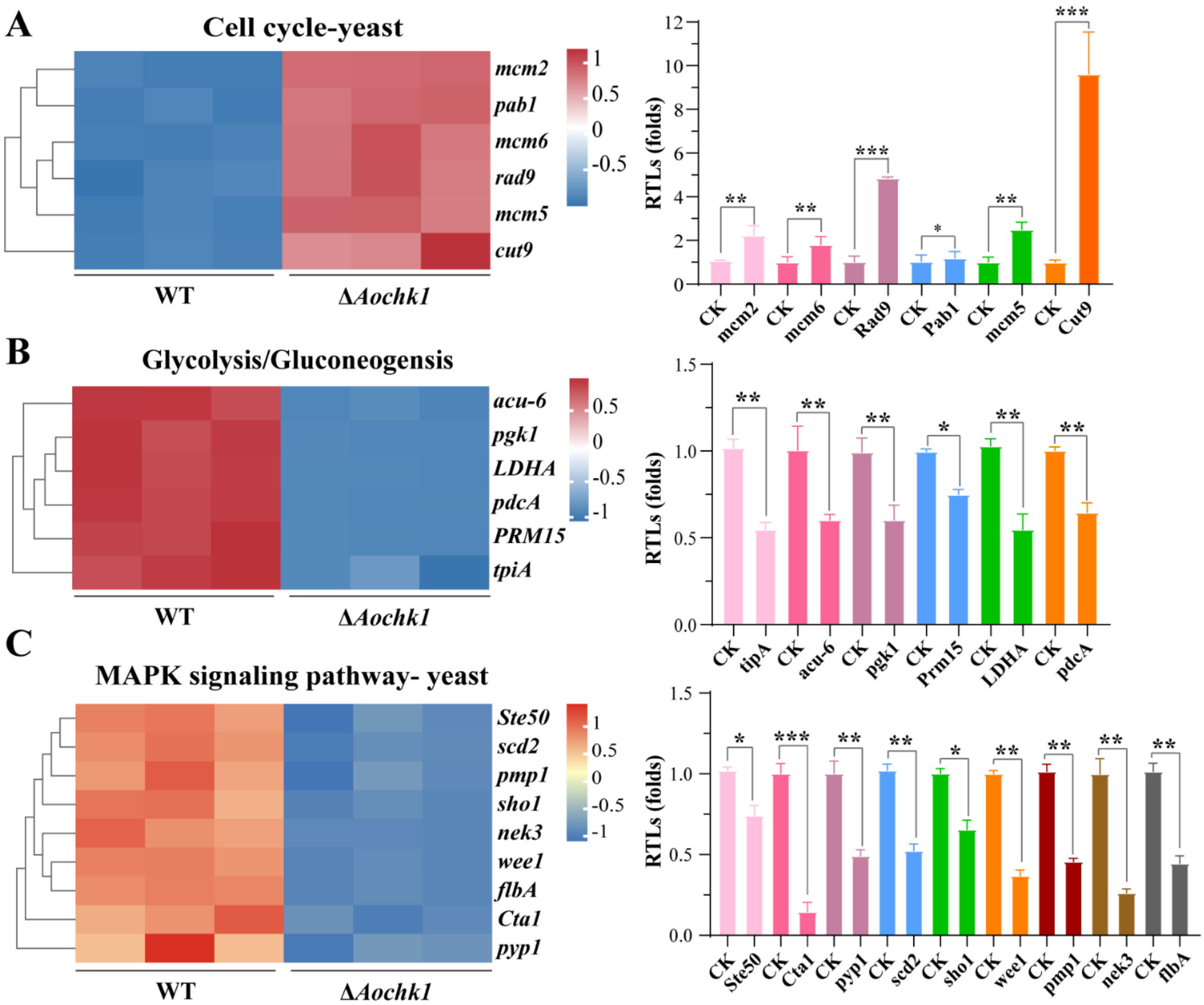


**Figure S2.** RT-qPCR analysis validated the transcriptome data of the wild-type and Δ*Aochk1* mutants. (**A-C**) Cluster analysis and RT-qPCR validation of related genes involved in the cell cycle (yeast) (**A**), Glycolysis/Gluconeogenesis (**B**), and MAPK signaling pathway (yeast) **(C)** pathways. (Tukey's HSD test: **p* < 0.05, ***p* < 0.01, ****p* < 0.001)

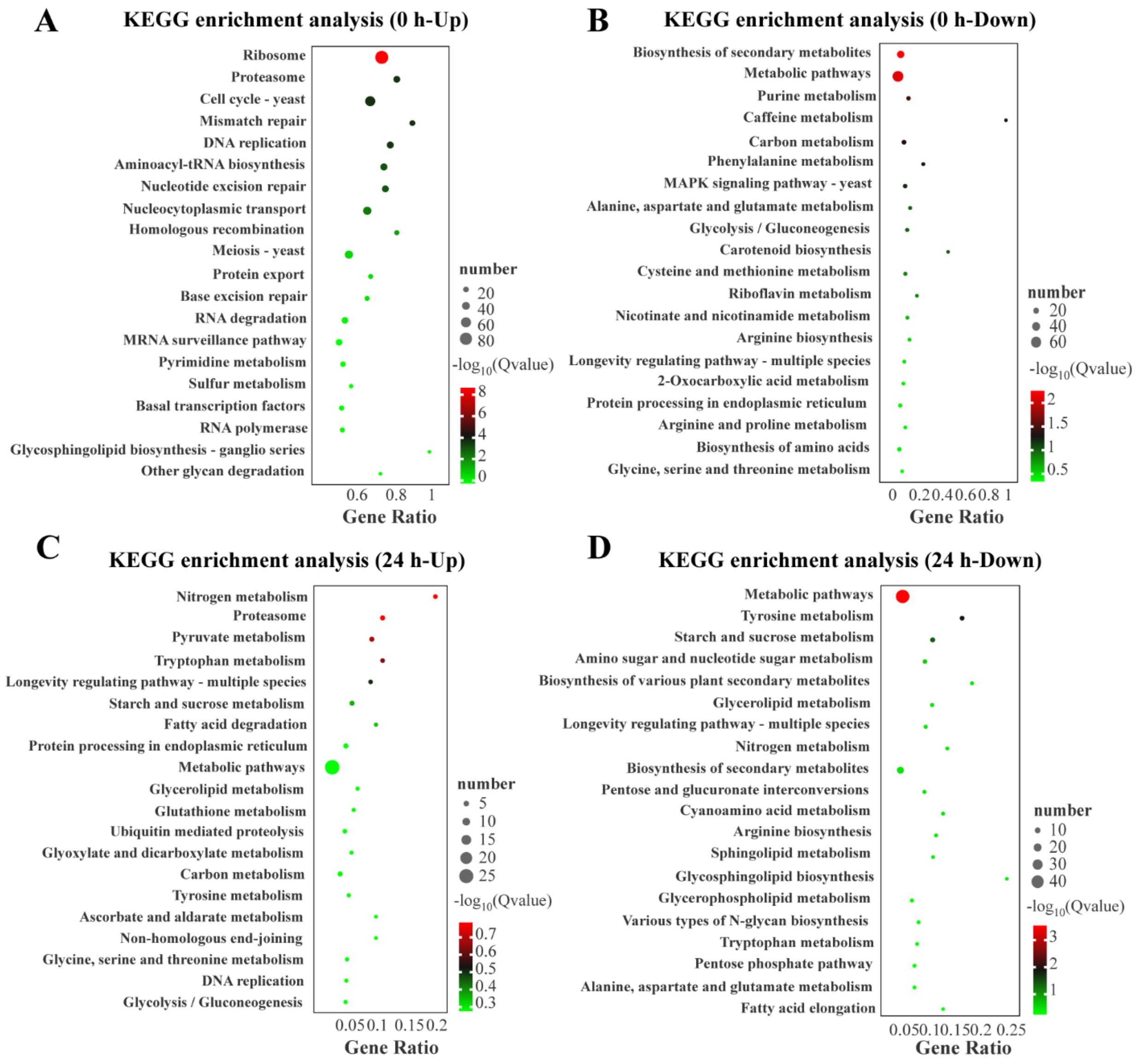


**Figure S3.** KEGG pathway enrichment analysis of differentially expressed genes (DEGs). (**A**) Enrichment analysis of up-regulated DEGs at 0 h post-induction. (**B**) Enrichment analysis of down-regulated DEGs at 0 h post-induction. (**C**) Enrichment analysis of up-regulated DEGs at 24 h post-induction. (**D**) Enrichment analysis of down-regulated DEGs at 24 h post-induction.

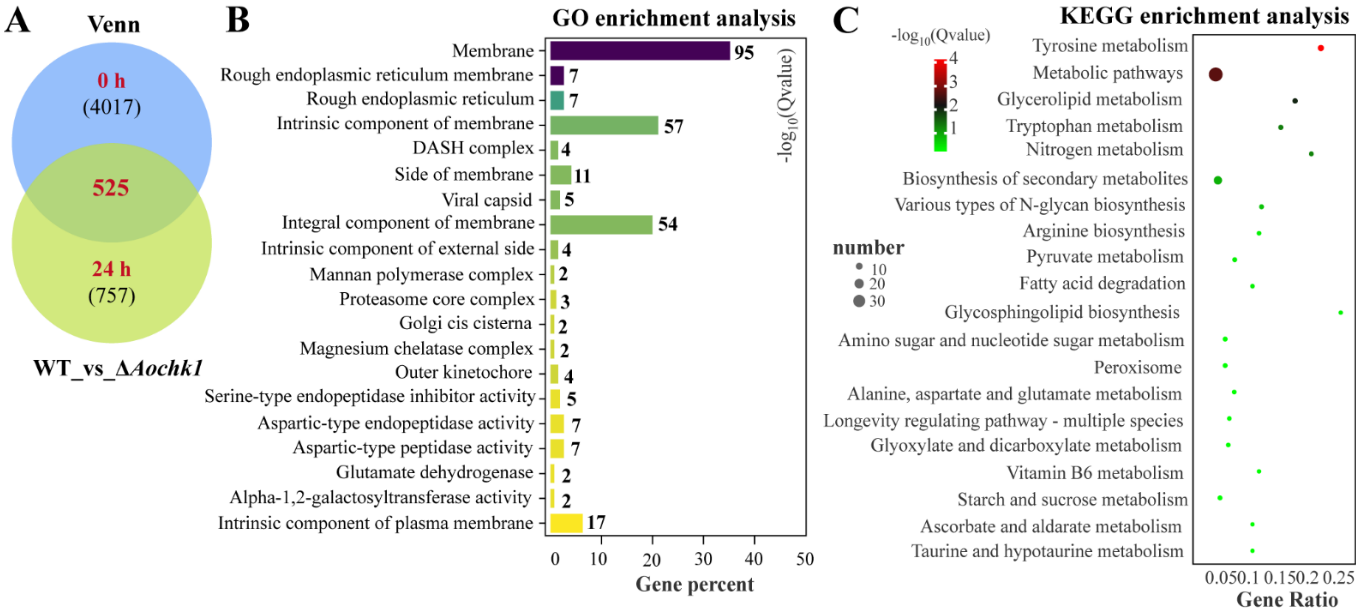


**Figure S4.** Analysis of trap-associated transcriptomic data. (**A**) Venn diagram of differentially expressed genes (DEGs) in WT vs. Δ*Aochk1* strains at 0 h and 24 h post-induction. (**B**) GO enrichment analysis of 757 consensus DEGs from the Venn diagram. (**C**) Bubble plot of KEGG pathway enrichment for the 757 consensus DEGs.

**Table S1.** Primers used for genetic manipulation in this study.

| **Primers** | **Primer sequence** |
|---|---|
| **Amplify the 5' flank of *Aochk1* gene** | |
| *Aochk1*-5f | GTAACGCCAGGGTTTTCCCAGTCACGACGCACGATTGAATAACCACG |
| *Aochk1*-5r | ATCCACTTAACGTTACTGAAATCTCCAACAGAGTTTCCGATACCAGAT |
| **Amplify the 3' flank of *Aochk1* gene** | |
| *Aochk1*-3f | CTCCTTCAATATCATCTTCTGTCTCCGACCAGTGAAGAAGGAGACGGAC |
| *Aochk1*-3r | GCGGATAACAATTTCACACAGGAAACAGCCTCAGGACGACGGATGGT |
| **Verify the transformants** | |
| *Aochk1*-f | TCAGAAGCCGAAGACAGT |
| *Aochk1*-r | TTCCACGATGAGGAGTTT |
| **Amplify *hph* resistance gene** | |
| *hph*-f | GTCGGAGACAGAAGATGATATTGAAGGAGC |
| *hph*-r | GTTGGAGATTTCAGTAACGTTAAGTGGAT |
| **Verify the transformants by RT-qPCR** | |
| RT-*Aochk1*-f | TTCCAACAACTTATCGCCGGTA |
| RT-*Aochk1*-r | TCGCCTATGTAACCTTTGCCTT |

**Table S2.** List of primers used for gene manipulation and RT-qPCR analysis in this study.

| Gene name | Sequence (5 F) | Sequence (3R) |
|---|---|---|
| **Sporulation-related genes** | | |
| AOL_s00083g25 (*stuA*) | AGCTCCCGAAACGAGTCTAA | ATTGATCATGTGATTATCCT |
| AOL_s00080g63 (*abaA*) | AACTTTATGCGCCTTGTCGT | TTGGCTAGGTGGTCTGTACG |
| AOL_s00210g120 (*medA*) | TCCGGCCCAATGATTCAGAA | AGATCGCAGGAACATGGTGA |
| AOL_s00083g487 (*lreA*) | TTCTCTTCGTCCCAAGCCAC | ACCGGTTCGAGTGGAGTCTA |
| AOL_s00080g93 (*lreB*) | CCAGGGTCGTCAGTATCTT | CAGCATCTTCCAGGTCAA |
| AOL_s00007g157 (*flbC*) | CTCTCCGGCAAAGACAATCG | GTCGACTGAGGATAGTAGCT |
| AOL_s00173g221 (*wetA*) | TTACATGCCACCCCAAGTCC | CAATTGCAACTGCGTCCACA |
| AOL_s00097g514 (*brlA*) | TTGAGGCCTCGATCCGTAGA | AGGTAGATGGCGCTGTTACG |
| AOL_s00076g640 (β-tublin) | CCACCTTCGTCGGTAACTC | TCGTCCATACCCTCACCAG |
| **Cell cycle related genes** | | |
| AOL_s00006g2 (*mcm2*) | ATTGCCAATCTCGAGGACCTTT | GCGGCATCTATAAGATCCCACA |
| AOL_s00007g146 (*pab1*) | TCGCATTTTCTCCTCTCAACCA | TCTCAGCGACGACTTTCAAAGA |
| AOL_s00006g301 (*mcm6*) | CCTTAGGAGAATGGTGCACAGT | ATTGTGTTCCATCAGCGGTACT |
| AOL_s00006g428 (*rad9*) | TCATTCATCTGGGCAGCTTCTT | GATGGGTAGAGTAATCTGCCGG |
| AOL_s00007g395 (*mcm5*) | GTACTAAAACAGGCCGGGAAGA | AACTTGTAGCGCCCTATCCAAA |
| AOL_s00007g409 (*cut9*) | CAAATACGAAGAAGCGCTCCAG | CCTCATTAGCTCGAGATGTCCC |
| **Glycolysis/Gluconeogensis** | | |
| AOL_s00004g362 (*acu-6*) | GAGAAGAACTCGAGCACTTCCA | TAACCATTTCCTTCTCGGCGAA |
| AOL_s00006g198 (*pgk1*) | GTACTCTCTTGCTCCTGTTGCT | CGATGTGGAATCGCAAGTTCTC |
| AOL_s00054g956 (*LDHA*) | AGCCATCCTCTTTGATCAACGT | AAGCGGTATTTGAGTGACGACT |
| AOL_s00215g149 (*pdcA*) | CGACTACTGCTGCGAAGGATAT | TTCCAGGTTAATCGGGGTCTTG |
| AOL_s00054g465 (*Prm15*) | ATGGGATTAGGAGGTTGGGAGA | TGGCAAAACCGGTATCCCATTA |
| AOL_s00004g442 (*tpiA*) | ATTGGTGAGACTCTCGAGGAGA | TTCCTGAGCTTGCTCAGTAGTG |
| **MAPK signaling pathway** | | |
| AOL_s00004g530 (*Ste50*) | AAGTCAAGATTGCCCATGAGGT | CACTGCGGATATCAATTGCTGG |
| AOL_s00006g411 (*Cta1*) | TCTCATGCTTCTCCAAGACACC | TAACCTCAAACTCTCCCCATGC |
| AOL_s00169g50 (*pmp1*) | TCGGAGAGGTAGTGATCAAGGT | ATGCTATTCTCTTCTGCGCGTA |
| AOL_s00078g396 (*sho1*) | ACCGCGCAAAGGCTATTTATTC | GCAATACCTGTTTCGCCAGTTT |
| AOL_s00176g88 (*nek3*) | AGGGAACATTGGGGAGGATAGA | TCGATACATTCCCATAGCTGCC |
| AOL_s00083g305 (*wee1*) | GGGACCCAAATCTGGAAGCTAA | AGTTGGGAAGAAGCCTGAGATG |
| AOL_s00215g516 (*flbA*) | ACTTTGGCCTATGCTTACGGAA | TTTGCAACAGCTCGAGTCATTG |
| AOL_s00043g69 (*pyp1*) | AGAGAAGGTTGGCAAGGAGATG | GTTTGGAGAGGCCCTATGTCAA |
| AOL_s00054g73 (*scd2*) | CCCGAGACTACAAACCCGTAAA | GCCTCGTAATAATCGTCCTCGT |

**Table S3.** Statistical analysis of quality control data of 12 sequencing samples.

| Sample | RawDatas | clean_reads | Q20 (%) | Q30 (%) | GC (%) |
|---|---|---|---|---|---|
| WT-0 h-1 | 41456340 | 41454228 | 99.27% | 97.48% | 47.93% |
| WT-0 h-2 | 38992418 | 38990062 | 99.08% | 96.89% | 47.94% |
| WT-0 h-3 | 37493868 | 37491854 | 99.24% | 97.40% | 47.88% |
| WT-24 h-1 | 45345236 | 45342214 | 99.33% | 97.69% | 49.19% |
| WT-24 h-2 | 44285238 | 44282340 | 99.28% | 97.52% | 49.16% |
| WT-24 h-3 | 43694716 | 43691892 | 99.34% | 97.75% | 49.15% |
| chk1-0 h-1 | 43177458 | 43174814 | 99.31% | 97.65% | 48.39% |
| chk1-0 h-2 | 44451828 | 44448922 | 99.26% | 97.49% | 48.43% |
| chk1-0 h-3 | 41644762 | 41642170 | 99.19% | 97.25% | 48.40% |
| chk1-24 h-1 | 37905076 | 37902692 | 99.27% | 97.49% | 48.70% |
| chk1-24 h-2 | 48512936 | 48509992 | 99.32% | 97.65% | 48.79% |
| chk1-24 h-3 | 40342342 | 40339412 | 99.27% | 97.50% | 48.83% |
